# FASTENER Feature Selection for Inference from Earth Observation Data

**DOI:** 10.3390/e22111198

**Published:** 2020-10-23

**Authors:** Filip Koprivec, Klemen Kenda, Beno Šircelj

**Affiliations:** 1 Jožef Stefan Institute, 1000 Ljubljana, Slovenia; filip.koprivec@ijs.si (F.K.); beno.sircelj@ijs.si (B.Š.); 2 Faculty of Mathemathics and Physics, University of Ljubljana, 1000 Ljubljana, Slovenia; 3 Institute of Mathematics, Physics, and Mechanics, 1000 Ljubljana, Slovenia; 4 Jozef Stefan International Postgraduate School, 1000 Ljubljana, Slovenia

**Keywords:** feature selection, machine learning, earth observation, genetic algorithm, information theory

## Abstract

In this paper, a novel feature selection algorithm for inference from high-dimensional data (FASTENER) is presented. With its multi-objective approach, the algorithm tries to maximize the accuracy of a machine learning algorithm with as few features as possible. The algorithm exploits entropy-based measures, such as mutual information in the crossover phase of the iterative genetic approach. FASTENER converges to a (near) optimal subset of features faster than other multi-objective wrapper methods, such as POSS, DT-forward and FS-SDS, and achieves better classification accuracy than similarity and information theory-based methods currently utilized in earth observation scenarios. The approach was primarily evaluated using the earth observation data set for land-cover classification from ESA’s Sentinel-2 mission, the digital elevation model and the ground truth data of the Land Parcel Identification System from Slovenia. For land cover classification, the algorithm gives state-of-the-art results. Additionally, FASTENER was tested on open feature selection data sets and compared to the state-of-the-art methods. With fewer model evaluations, the algorithm yields comparable results to DT-forward and is superior to FS-SDS. FASTENER can be used in any supervised machine learning scenario.

## 1. Introduction

Land cover classification based on satellite imagery is one of the most common and well-researched machine learning (ML) tasks in the Earth Observation (EO) community. In Europe, the Copernicus Sentinel-2 missions provide large amounts of global coverage EO data. With 5-day revisit times, Sentinel-2 generated a total of 9.4 PB of satellite imagery by April 2020 [1]. The total amount of EO data available through the Copernicus services is estimated to exceed 150 PB. The data represent an opportunity for building solutions in various domains (agriculture, water management, biology or law enforcement); however, computationally efficient methods that yield sufficient results are needed to deal with this big data source. The most important issue when trying to maximize the accuracy of statistical learning methods is effective feature engineering. With many features, the algorithms become slower and consume more resources. In this paper, we present FASTENER (FeAture SelecTion ENabled by EntRopy, https://github.com/JozefStefanInstitute/FASTENER) genetic algorithm for efficient feature selection, especially in EO tasks.

EO data sets are characterized by a large number of instances (millions or even billions) and a fair number of features (hundreds). These characteristics differ from the usual feature selection data sets that often contain up to several hundred instances and usually thousands of features. FASTENER exploits entropy-based measures to converge to a (near) optimal set of features for statistical learning faster than other algorithms. The algorithm can be used in many scenarios and can, for example, complement multiple-source data fusion and extensive feature generation in the fields of natural language processing, DNA microarray analysis or the Internet of Things [2]. FASTENER has been tested on a land cover classification EO data set from Slovenia. Additionally, we have tested the algorithm against 25 general feature selection data sets.

The main evaluation use case presented in this paper is based on our previous contributions [3,4] regarding crop (land cover) classification using Sentinel-2 data from the European Space Agency (ESA). The scientific contributions of our work are as follows:

1. A novel genetic algorithm for feature selection based on entropy (FASTENER). Such an algorithm reduces the number of features while preserving (or even improving) the accuracy of the classification. The algorithm is particularly useful for data sets containing a large number of instances and hundreds of features. A reduced number of features reduces learning and inference times of classification algorithms as well as the time needed to derive the features. By using an entropy based approach, the algorithm converges to the (near) optimal subset faster than competing methods.
2. Improvement of the state-of-the-art in feature selection in the field of Earth Observation. FASTENER yields better result than current state-of-the-art algorithms in remote sensing. The algorithm has been tested and compared to other methods within the scope of the land-cover classification problem.
3. Usage of pre-trained models for information gain calculation for feature selection in Earth Observation scenarios. Usually, it is computationally expensive to train a machine learning model. The inference phase is much faster. FASTENER exploits the pre-trained models in order to estimate the information gain of certain features by inferring from data sets with randomly permuted values of the evaluated feature. This approach improves the convergence speed of the algorithm.

The paper is structured as follows. In the next section, related work regarding feature selection algorithms as well as state-of-the-art in feature selection in Earth Observation is described. Section 4 contains a thorough description of the data used in the experiments and describes our algorithm. Section 5 describes while Section 6 discusses the experimental results and compares FASTENER to other algorithms based on NSGA-II. Finally, our conclusions are presented in Section 7.

## 2. Related Work

When tackling problems related to EO, quite often the older and simpler algorithms (ReliefF) have given the best results. This section presents the current state-of-the-art in feature selection and particularly its applications within the EO community. It also describes supporting fields of the work presented in this paper, such as the current status in land-cover classification and the multi-objective optimization approach to feature selection.

### 2.1. Multi-Objective Optimization and Its Usage in Feature Selection

Data science often reduces its evaluation to a single score (or fitness function) that explains the accuracy or efficiency of a particular methodology. There are other limitations that should be considered. On the one hand, there are the available resources (e.g., memory, processors, space), while on the other hand the response time should be considered (i.e., the preferred response time for a particular methodology). Quite often, the most appropriate approach should minimize the need for resources and response time while maintaining sufficient accuracy. The multi-objective optimization [5] strives to find a set (or Pareto front) of solutions (in our case feature sets) that achieve the best possible classification accuracy with a limited number of features.

Multi-objective optimization methods have already been used for feature selection purposes, although their usage in the field is limited due to their computational complexity [6]. The classical NSGA-II algorithm [5] was used for feature subset optimization in [7]. NSGA-II uses fast non-dominated sorting for selecting viable candidates for the next generation parent population. The methodology has been improved by the usage of Reduced Pareto Set Genetic Algorithm with Elitism (RPSGAe) [8] for achieving a more efficient directed search in the feature space [9]. The elite-preserving operator suppresses the deterioration of the population fitness along with the successive generations. RPSGAe reduces the number of solutions on the efficient (Pareto) front while maintaining its characteristics intact, similarly to the purge function in the FASTENER algorithm. NSGA-II feature selection approach has also been extended in [10] with six different importance measures, including representation entropy which is based on information theory. The research shows that there is no preferred importance measure that would be a good fit for all feature selection problems. FASTENER uses importance measures that are based on real model evaluations and are therefore more reliable than estimates based on the features themselves.

Pareto-optimal subset selection (POSS) [11] is also a genetic algorithm, but it only uses mutation techniques (while NSGA-II also uses cross-over methods). Other methods explore several variants of directed searches within the feature space to converge to the sub-optimal data set faster (e.g., FS-SDS [12] uses stochastic diffusion search and extreme learning machine [13] as an embedded classifier). Other methods, such as forward selection and backward elimination use an exhaustive step-by-step search to find the most optimal feature set [14]. These methods are much slower (and quite often do not converge to a small-enough data set within a reasonable time) and could even converge to a local minimum.

Our approach extends POSS and NSGA-II algorithms and suggests the usage of entropy-based indices based on previously trained models to steer the iterative feature selection process. The parameterized mutation strategy introduces additional features to the candidate feature sets with respect to changes in the Pareto front. The Pareto front is periodically purged in order to eliminate non-suitable candidates from the loop. With these improvements, FASTENER converges to a (nearly) optimal solution faster than the compared methods.

### 2.2. Feature Selection and Dimensionality Reduction for EO Tasks

The automatic spectro-temporal feature selection (ASTFS) [15] workflow provides a search over the feature space by incrementally extending the feature set with effective features (those that improve classification scores) ordered by their global separability index [16]. A similar approach is implemented in [17], where the authors use mutual information (MI) [18] and Fisher’s criterion to select the *k* most important features. Mutual information based on entropy estimates from *k*-nearest neighbour distances [18] is data-efficient and has a minimal bias. The problem of estimating MI between discrete and continuous features has been solved in [19], which can be applied to classification tasks (discrete) with continuous features. Although the separability index and mutual information provide good heuristics for adding different features, the non-iterative (non-wrapping) algorithms do not necessarily yield the optimal feature set. Our algorithm offers a more thorough search, which is controlled by the genetic nature of the algorithm. The approach has been tested using timeless features for land cover classification [20,21].

A revision and test of multiple feature selection algorithms (similarity-based, statistical, sparse learning, information theoretical, and wrapper methods) [22] showed that ReliefF (a similarity-based approach) [23], although not among the recent favourites, yielded the best results in a particular scenario of parthenium weed infestation detection.

Our algorithm can be applied to any feature selection scenario as it is expected to converge to the (near) optimal subset selection. FASTENER is expected to perform at least as good as any other similarity-based approaches; however, several iterations would be required during the learning process to find the (near) optimal subset of features.

Genetic algorithms have been used in EO scenarios for feature selection of hyper-spectral EO images [24]. In contrast to multi-spectral (as in our case), hyper-spectral images contain a significantly larger number of bands. The approach in [24] does not use any entropy-based features to optimize the search and is, in that respect, similar to POSS. The latter has been tested in land cover classification [4] and yielded good results.

In an EO environment, where data indices are abundant, the calculation of some features is computationally very intensive and external data are difficult to acquire (weather data, soil samples). Reducing the number of features has a significant positive impact on the overall algorithm performance in a real-world scenario. As the production of global EO products sometimes take thousands of computing hours, efficient feature selection algorithms could reduce the costs by 50–95%.

### 2.3. Land Cover Classification and Feature Engineering

There are essentially three approaches to land cover classification based on EO data [25]. The first approach is object-based and focused on a single image, while the other two approaches are pixel-based. From the latter, a simpler approach is based on single-image data, while the other is based on a time-series of images. Single image approaches depend on the current situation (e.g., cloud cover), whereas time series based approaches inherently overcome this shortcoming. Sentinel-2 mission from the Copernicus programme is dedicated to agriculture and their satellites re-visit times are 5 days. It is possible to construct an interpolated time-series of different EO sensors throughout the entire vegetation period [3]. A simple feature extraction involves taking particular sensor values and derived features at pre-selected points in time and combining them into a feature vector. A more intelligent approach involves the extraction of timeless features such as the maximum value of a particular time series, length of the maximum interval, steepness of the steepest slope and others [20,21]. Our evaluation of the FASTENER algorithm is based on the latter approach [3].

The best accuracy scores in land cover classification were achieved with deep learning [26]. These approaches reduce the value of feature engineering that originates mostly from domain experts and/or extensive experimental knowledge. Deep neural networks are able to derive the important features exclusively from raw data. Of course, the data must be extensive, which is the case in EO. However, this comes with a price; namely, extreme computational requirements reduce the usability of these methods.

Our goal is to develop lean EO machine learning models that are capable of capturing most of the information with limited resources. Intelligent feature selection, together with smart feature engineering and a pragmatic choice of the learning models is one of the three pillars of smart EO.

## 3. FASTENER—A Genetic Algorithm for Feature Selection

The presented algorithm is based on the POSS genetic algorithm and the Pareto ensemble pruning proposed in [27,28]. The main objective of the algorithm is to select an optimal subset of available features from a large feature set (N>100). The problem could be represented mathematically as finding the optimal (or near-optimal) binary vector *x* of an indexed set ${\left\{0,1\right\}}^{N}$. Finding the optimal subset is NP-complete. Each element in *x* corresponds to a particular feature that is included or excluded in our decision model. The optimality of such a set is measured by a scoring function, which we will denote by A(M;x) (accuracy measure of model *M* on a subset of features *x*). Some standard examples of such measures in machine learning classification problems include precision, recall, accuracy and $F_{1}$ score. In our experiments, the $F_{1}$ score was used. We also adopt the notation |x| to denote the number of bits set in *x* that directly corresponds to the number of selected features. Mathematically, for a fixed classification model *M* and the number of features *k*, we search for such an *x* where the following maximum is reached:

$$\max_{\begin{array}{c} x\in {\left\{0,1\right\}}^{N}\\ |x|=k \end{array}}A\left(M;x\right).$$

As in POSS, the function x↦(|x|,A(M;x)) creates a two-dimensional Pareto front. In one dimension, we evaluate *k*, the number of selected features, and in the other dimension, we present *A* score, which represents the optimality (e.g., accuracy) of such a subset. In this space, the ordering of instances is defined as follows. The pair $\left(k_{1},s_{1}\right)$ dominates the pair $\left(k_{2},s_{2}\right)$ if and only if $k_{1}\leq k_{2}$ and $s_{1}\geq s_{2}$. Intuitively, the smaller set (by cardinality) of features provides classification results, which are at least as good as for the larger set. We denote such a relation by $\left(k_{2},s_{2}\right)⪯\left(k_{1},s_{1}\right)$. The relation is obviously transitive but does not induce linear ordering. Pairs that are not comparable through such a relation are of particular interest, as they lie on the Pareto front. Feature subsets that lie on the Pareto front (not strongly dominated by other subsets) are the desired subsets. They provide the best classification power using the smallest number of features. The final result of a feature selection algorithm is the final Pareto front. The final Pareto front can be seen as an accumulation of the best results for a fixed *k*. The features with the best performance can then be selected from the front automatically (with a certain cut-off threshold), manually or by using additional information (e.g., time to calculate the features). The results clearly show that the Pareto front “plateaus” after the inclusion of some features and this fact can easily be used to automatically select the best number of features.

Our modification of the POSS algorithm combines Pareto front searching with a genetic algorithm to incorporate additional statistical information when recombining genes. The main ingredient in the algorithm is the concept of an *item*; a subset of features and *scored item*, which is an item with an assigned score corresponding to such a subset of features. The size of an item denoted by |ITEM| corresponds to the number of features selected. The algorithm works by successively evaluating items, combining them, and updating the Pareto front.

Each subset of features can be represented directly as a binary number, where set bits correspond to the included (selected) features and unset bits correspond to excluded ones. This allows for a natural representation of genes for such an item with a binary string or bit-set and also gives a natural human-readable representation as an arbitrary sized integer. Apart from this, many later needed operations can be quickly implemented as simple operations on a binary string. The size of an item corresponds to the number of set bits, the bit-wise and operation between two items corresponds to the genes that are common to both of them, while the bit-wise xor returns all different selected features.

In our implementation, the integer representation of item genes is treated in a little-endian way, as this allows easier extension of feature sets and keeps integer representations valid even when adding new features. For example, in a set of six features with 32 possible feature combinations, binary string 11010 represents a subset containing features with indices 0, 1 and 3 and is represented by a decimal number 11. Even if the number of features is increased, such a subset would still be represented by the number 11, albeit its binary string representation would be extended by zeros on the right side.

**Figure 1:**
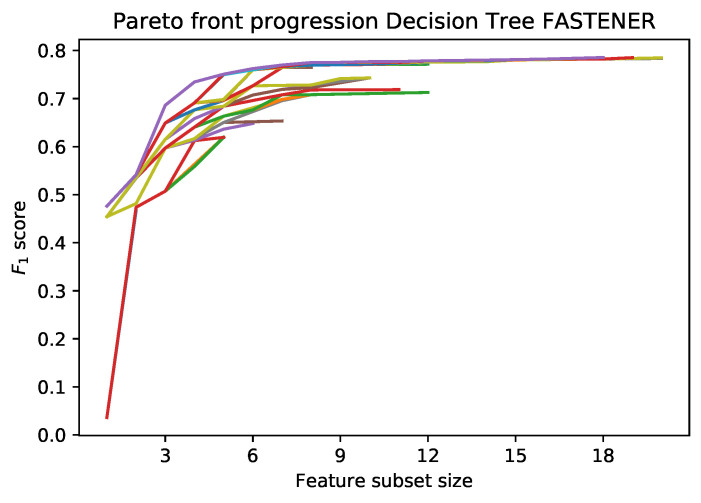
Iterative improvements of Pareto front from the first generation. Each next generation’s Pareto front achieves better $F_{1}$ score.

An example of the incremental Pareto front update is shown in Figure 1. Each subsequent generation of the FASTENER algorithm produces a Pareto front that dominates the Pareto fronts from previous generations (in rare cases, where no improvement is made, the Pareto fronts of two subsequent generations may be the same). The main objective of the algorithm is to converge as quickly as possible to a theoretically limiting front (in terms of fitness function evaluations).

### 3.1. Algorithm

The algorithm stores the current Pareto-optimal items and progresses in generations. For each k≤N it keeps an item with the best score *A*. If there is no such item this means either that it is strictly dominated by another item or that the algorithm has not yet evaluated an item this size. In our implementation, the Pareto front is implemented as a simple dictionary. Apart from the Pareto front, the current population is kept in a separate set. The decoupling of the current population from the Pareto front allows the algorithm to “explore” different combinations more easily, while the Pareto front continues to be updated at each iteration.

**Figure 2:**
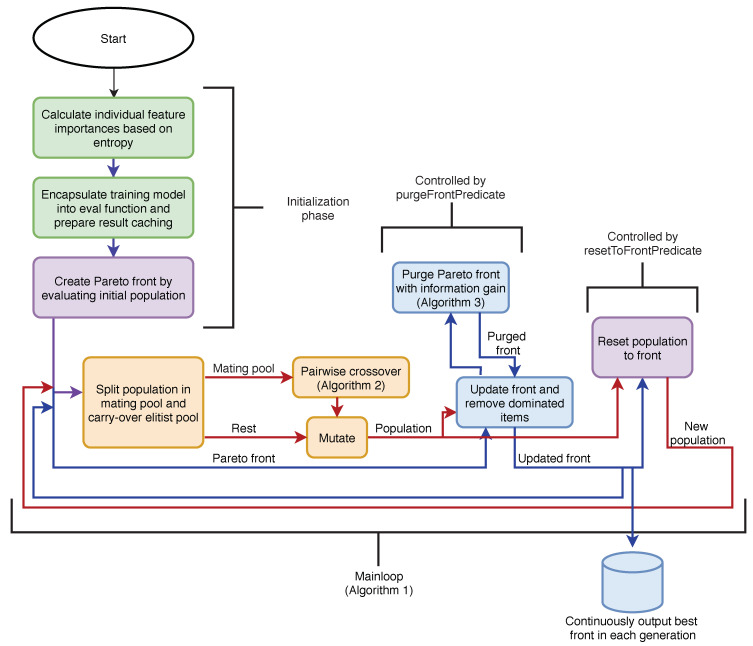
The flow diagram of the FASTENER graphically represents Algorithm 1 and puts Algorithms 2 and 3 into context. In the figure, red/orange colour objects represent population-based operations, while blue colour objects represent Pareto front related operations. The violet colour represents both, the population and the Pareto front. Green boxes refer to technical details of the algorithm. The algorithm includes the initialization phase and the main loop. In the initialization phase, the initial population is prepared and evaluated and the technical prerequisites for the algorithm are created. In the main loop, the population is first split into a mating pool and a (gene) carry-over elitist pool. The mating pool first enters the crossover phase based on Algorithm 2 and is being mutated together with the elitist pool. Then the new Pareto front is updated and purged (Algorithm 3). Finally, the main loop is closed by registering the new population as the next generation Pareto front.

As usual, in each generation, the current population of items is mated, a random genetic mutation is introduced and newly acquired items are evaluated. For each evaluation, the Pareto front is updated. If a new item is strictly better than an item on the front with the same size, the newly evaluated item is placed on the Pareto front. After all new items have been evaluated, the front is purged by removing items that are strictly dominated by other items. The pseudo-code for the basic algorithm is presented in Algorithm 1. The algorithm’s flow diagram is depicted in Figure 2.

To prevent the search phase of the algorithm from diverging (the population as a whole is not controlled by the scoring function), the population is periodically reset to the current Pareto optimal items. In the presented algorithm, this decision is based only on the generation number; however, our implementation also considers the rate with which the running Pareto front is being updated between generations.
**Algorithm 1** Basic algorithm**Require:**Initial population popEvaluation function evalFNumber of iterations KMating pool selection strategy selectPoolCrossover strategy crossoverMutation strategy mutatePredicate resetTOFrontPredicate purgeFrontPredicateFront purging procedure purgeFront1:**function** FeatureSubsetSelection2:    front ← pop3:    **for** gen = 1 to K **do**4:        pool ← selectPool(pop)5:        pool ← crossover(pool)6:        pop ← mutate(pop ∪ pool)7:        pop ← evaluate(pop, evalF)8:        front ← front ∪ pop                       ▹ Add evaluated population to Pareto front9:        front ← removeDominated(front)      ▹ Remove unoptimal items from Pareto front10:        **if** purgeFrontPredicate(gen) **then**11:           front ← purgeFront(front)12:        **end if**13:        **if** resetToFront(gen) **then**14:           pop ← front15:        **end if**16:    **end for**17:    **return** front18:**end function**

### 3.2. Mutation

An important part when considering gene manipulation and modification is to keep the overall objective in mind. We want to find the optimal subset of features with good accuracy characteristics. The ultimate goal is to obtain a subset with a size much smaller than *N*. In preliminary experiments, the number of features with a sufficiently predictive power was around $\sqrt{N}$, which means a significant reduction in both training time and in data preparation time (which is true especially for EO data sets). Mutation and crossover methods should therefore be tailored to help maintain, reduce or only slightly increase the size of items.

The most interesting parts of the algorithm are the crossover and mutate procedures. The procedure for the mutation is adopted from the POSS algorithm and is a simple random bit mutation. Each random bit in the gene is flipped independently with a probability $\frac{1}{N}$. Thus a new item is generated from each of the items selected for mating and, most importantly, the expected value of set bits is kept approximately the same. Formally, the expected value of the number of flips is 1, and therefore the mutations gravitate slowly toward *x*-s where $\left|x\right|=\frac{N}{2}$. Since the desired range of features in which we can expect satisfactory score results is much lower than the asymptotic behaviour of mutations, this type of mutation serves as a size increaser. Mutation procedures therefore slowly increase and add new features to be considered. Furthermore, varying the probability of flipping is an easy way to control the speed of the introduction of new features. Setting the mutation rate to $\frac{a}{N}$ for a>1 and varying *a* with generation number and changes in the Pareto front updates can significantly increase the search space. In addition, all further analyses are still valid even if the mutation is set as an arbitrary function $f:N^{+}\to \left(0,1\right]$ only dependent on *N* and heuristic data about mutations (independent of bit-index and bit status), called *mutation function*.

### 3.3. Crossover

The crossover procedure is designed to reduce the size of the newly produced item. The mating pool selection used for the algorithm is a simple random mating pool with a fixed size. Two different items from the mating pool are combined using the crossoverPair procedure. The crossoverPair procedure itself is presented in Algorithm 2 and requires 4 input parameters: two items to be mated, a scaling function for the number of non-intersected genes, and a set of entropy information for each individual feature. Without loss of generality, we may assume that |ITEM1|≥|ITEM2|.

**Lemma** **1.** *Let ITEM1 and ITEM2 be a random independent subset of ${\left\{0,1\right\}}^{N}$ where the probability of each gene being set is some positive function $f:N^{+}\to \left(0,1\right]$. The expected size of an intermediate gene conditional on the sizes of its parent genes is*

$$E\left[|\mathrm{ITEM}1\cap \mathrm{ITEM}2|||\mathrm{ITEM}1|=a,|\mathrm{ITEM}2|=b\right]=\frac{ab}{N}=\frac{\left|\mathrm{ITEM}1||\mathrm{ITEM}2\right|}{N}$$

We can similarly derive conditional expectation for size change.

**Lemma** **2.** *The probability of an *item* having size k, assuming that each feature is selected independently with probability $f:N^{+}\to \left(0,1\right]$ is*

P(|ITEM|=k)=Nk(1−f(N))N−kf(N)kNf(N).

**Lemma** **3.** *The probability of an intermediate item having size k conditional on sizes of its parents is similarly*

P(|A∩B|=k|∣|A|=a,|B|=b)=NkN−ka−kN−ab−kNaNb.

*Since all features are equally likely to be selected, conditional probability is independent of scaling function f.*

**Lemma** **4.** *By linearity of expectation and assumption that |ITEM1|=a and |ITEM2|=b we can easily derive*

$$E\left[max\{|\mathrm{ITEM}1|,|\mathrm{ITEM}2|\}-|\mathrm{ITEM}1\cap \mathrm{ITEM}2|||\mathrm{ITEM}1|=a,|\mathrm{ITEM}2|=b\right]=\left|\mathrm{ITEM}1\right|\left(1-\frac{\left|\mathrm{ITEM}2\right|}{N}\right).$$

**Remark** **1.** *The assumption that each feature is selected independently with probability f(N) should not be confused with each subset of features selected with equal probability. In our case, this is beneficial because smaller sets are intrinsically better, which are easily controlled by the mutation function f.*

**Algorithm 2** Randomized crossover with information gain weighting
**Require:**

First item item1Second item item2Scaling function for number of genes onGeneScalingIndividual feature entropy informationEntropy
1:**function** crossooverPair2:    **if** |item1| < |item2| **then**3:        swap(item1, item2)4:    **end if**5:    intermediate ← item & item2             ▹ Intersection of genes6:    rest ← item1 ⊕ item2       ▹ Bitwise XOR, features present in exactly one item7:    addNum ← onGeneScaling(abs(|item1| - |item2|))8:            ▹ Scale the number of new features according to provided function9:    additional ← rSelect(addNum, informationEntropy, rest)10:         ▹ Randomly select addNum features, weighted by precalculated entropy11:    mated ← intermediate | additional            ▹ Add selected features12:    **return** mated13:
**end function**



**Figure 3:**
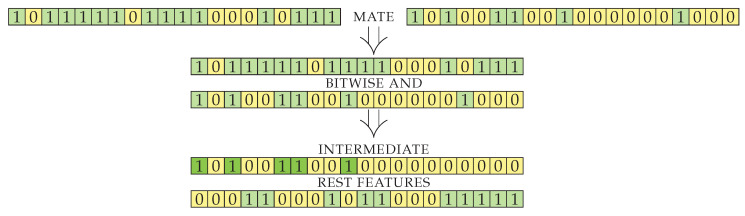
Schema of mating of 2 genes. With bit-wise and operation we produce the intermediate set and with bit-wise exclusive or xor we produce the rest (features) set.

After crossover, the resulting genes initially contain features that were selected in both parents. The intersection of features is a good starting point, as it seems to produce good results during the running of the algorithm. Since the implementation of the genes is a simple bit string, the intermediate result is simply bitwise logical and of its parents’ genes. Another way to combine the parents would be to produce offspring with the union of genes. The latter approach produces offspring that is too large (contains too many selected features) and is thus unsuitable for our objective, where we want to select a sufficiently small feature set. The size of the intermediate result obtained by the intersection (line 5) is smaller than or equal to the size of item2 (smallest of the parents), but can be much smaller, depending on the input sets (see Lemma 3). With the intersection, the number of genes decreases too rapidly (see Lemma 4) and disposes of useful information. We therefore use additional genes presented in exactly one of the parents (generated by element-wise exclusive or) to construct an additional set of features with good information gain. The visualization for the first part of crossoverPair procedure on an example case is shown in Figure 3. The genes of two items to be mated are split into two indexed sets: an intermediate set containing genes from both parents and a set called *rest* containing genes to be used for the enrichment of intersected genes.

Enrichment of the intermediate set of genes is done as follows. First, the number of enrichment genes is calculated using the onGeneScaling function. This function is used to control the size of the final offspring. From Lemma 4, it follows that the expected size of the rest (features) set, with the size of the largest parent being constant, is linear to the size of the smaller parent (item2). Adding all genes from the rest set will result in an offspring too large (and diverge toward the whole feature space being selected), selecting none of the genes will diverge towards very small sets. In our experiments, we used the function $x\mapsto ⎣\frac{x}{2}⎦+1$ (for onGeneScaling) that scaled the amount of features appropriately for our data set. For a larger number of features, the functions $\sqrt{x}$ and $\log_{a}x$ with a sufficiently small base *a* are a good choice. As with most parameters, onGeneScaling can be swapped during the run-time and made dependent on the conditions of front updates and statistics.

The second part of the mating algorithm selects the best performing features using heuristics based on information gain. From all features in the rest set, *addNum* are randomly selected (using a weighted random selection, where the weight of each feature is its individual precalculated information gain). The final result is an item with genes from the intersection set and selected genes from rest set. In the implementation, the results are simply combined with element-wise OR. The heuristics for the information gain in our algorithm is based on mutual information gain. The scikit-learn implementation [29] of mutual information gain is used for each feature in the train set.

**Figure 4:**
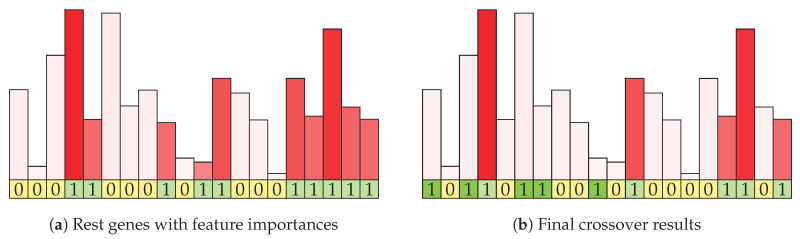
Visualization of the rest set and final mating result. Information gain of a particular feature is depicted by the height of the column above a particular feature.

Figure 4a shows the continuation of the example mating algorithm from the previous figure. Each of the initial features is represented with its information gain, which is shown by the height and intensity of the shade of the column above it. Columns for features in the rest set are shown in red (and the corresponding genes are green in the bottom row). Of the eight features in the rest features row, five are selected using weighted random function. Figure 4b shows the visualization of the result (offspring) of the mating procedure. The genes selected by intersection are marked with dark green shading, while the genes selected by information enrichment are shown in light green. The corresponding information gain statistics in shown with column color intensity. In this example, two genes of size 14 and 6 were combined and they produced a result with size 10, which may be further increased by the mutation strategy. The resulting item contains genes that are present in both parents and were therefore rated as good in the previous evaluation. It is enriched with a limited number of genes that are present in either parent, weighted according to their information gain.

### 3.4. Purging Features from Pareto Front Using Information Gain

An initial version of the algorithm presented in Algorithm 1 has been further improved to exploit pre-trained models for the calculation of the information gain and to purge non-relevant features from items on the Pareto front. For each item added to the front, a model trained on its genes is stored. Since the number of elements is small compared to the learning data, storing the models results in virtually no additional effort in run-time and is a useful tool for evaluating the optimization over time. In the same way as when the population is reset to the Pareto front, another predicate is introduced to indicate when the Pareto front should be purged using the information criterion. The gene purging procedure is presented in Algorithm 3.
**Algorithm 3** Gene purging algorithm**Require:**Current Pareto front frontEvaluation function with ability to randomly shuffle features evalF1:**function**purgeParetoFront2:    newFront = ← Dict.empty      ▹ Empty dictionary representing new Pareto Front3:    **for** item in front **do**4:        **if** |item| == 1 **then**5:           **continue**            ▹ Items with only one feature cannot be reduced6:        **end if**7:        baseResult ← item.result        ▹ Score acquired by using all features from item8:        scoreDecrease ← []             ▹ Score decrease for each feature9:        **for** geneInd in item.genes **WHERE** ISSET(item.genes[geneInd]) **do**  ▹ Check only set genes10:           newScore ← evalF(item.model, item.genes, geneInd)▹ Evaluate existing trained model on test data set, where values of feature geneInd are randomly shuffled11:           scoreDecrease[geneInd] ← baseResult - newScore12:        **end for**13:        bestInd ← argmin(scoreDecrease)         ▹ Take the gene which showed smallest score decrease when shuffled14:        newItem ← Item(genes=unsetBit(item.genes, bestInd))        ▹ Create new item where the gene with the smallest score decrease is unset15:        newFront[|newItem|] ← Evaluate(newItem, evalF)                  ▹ Evaluate newly created item (train and test model)16:    **end for**17:    front ← front ∪ newFront       ▹ Update current front with newly calculated items18:    front ←removeDominated(front)       ▹ Remove unoptimal items from Pareto front19:    **return** front20:**end function**

The main idea of purging the items on the Pareto front with information criterion is to discard unneeded features from items that already perform well. For each item on the Pareto front, we try to discard a feature that makes the least contribution to the item’s score. The obvious way to do this would be to transverse through all the features used in the prediction model, remove superfluous features one by one, and train and re-evaluate such a model. For each item, a new model must be trained and re-evaluated. This is time-consuming and offers only minor improvements. Our proposal is to use a heuristic approach to select a feature to be removed. For each such feature, an existing model corresponding to the item is used (saving time by avoiding the training of a new model) and evaluated according to the previously used test data set. For each selected feature, its values are randomly shuffled across the test data set. The motivation for such heuristics is that the shuffling values of a feature with strong predictive power has a much stronger effect on the resulting score than shuffling values of a feature with only low predictive power. In this way, we can provide heuristics for estimating the predictive power of features in a model-agnostic way using only a linear number of model calls. Such heuristics provide a noticeable acceleration and can be used for any type of model. The approach targets black box models with a high ratio between training and inference time/resource consumption—e.g., gradient boosting or complex neural networks. For each newly shuffled feature in an item, a change in the base score is recorded (line 11). Interestingly, sometimes the change can even be negative if the included features are detrimental to the overall prediction procedure. This is proving to be an effective method for quickly sorting out bad features introduced by mutations at the end of simulations when the exploration is limited to the feature space previously explored.

The feature whose shuffling has the most detrimental effect is taken and a new item is constructed whose genes are the same as those of the original element, except that the resulting feature is omitted (lines 13 and 14). This procedure can be further generalized. For example, first scoreDecrease could be scaled with some function (for example, $x^{3}$) and then the feature to be removed could be sampled by these new scoreDecrease weights from the set. Special care should be taken that the scaling function is monotonous (and preferably a bijection) since negative values can occur and should not be bundled with positive ones. If a random selection is used, the final probabilities must also be re-scaled correctly due to possible negative weights. After the feature to be removed is selected, the new item is evaluated and all new items are brought to the front. The front should also be purged to take into account possible new features on the front.

The most important heuristic optimization used by the front purging functions is based on the fact that only a new model is built with the features with the most promising importance score (as inferred by a model when feature values are shuffled). The time required for this operation is linear in the number of features. For each feature, the model requires one evaluation (inference) on the training set. The implementation of the gene purging can be further optimized so that each feature is selected only once. Under the reasonable assumption of sufficiently deterministic training and evaluation, it is easy to see that evaluating an item and (potential) addition of a feature to the front is an idempotent operation. Removing the same feature from the same item on the front several times is not a reasonable change and therefore another feature could be considered for removal. An appropriate threshold for reducing the score may also be introduced so that features with very high predictive power are never removed.

## 4. Data

FASTENER is focused on improving the state-of-the-art in EO land-cover classification. The description of the EO data set is given in Section 4.1 and Section 4.2. Apart from that, the algorithm was tested on 25 additional feature selection benchmark data sets with a varying number of features, label classes and instances, in order to be compared against the existing state-of-the-art methods.

### 4.1. EO Data

Earth Observation data were provided by the EU Copernicus program’s Sentinel-2 mission, whose main objectives are land observation, land use and change detection, support for generating land cover, disaster relief support and climate change monitoring [30]. The data comprise 13 multi-spectral channels in the visible/near-infrared (VNIR) and short wave infrared (SWIR) spectral range with a temporal resolution of 5 days and spatial resolutions of 10 m, 20 m and 60 m (the latter is used for diagnostics only) [3]. Sentinel’s Level-2A products (surface reflectances in cartographic geometry) were retrieved via the SentinelHub (https://www.sentinel-hub.com/) services and processed using the eo-learn (https://github.com/sentinel-hub/eo-learn) library. In addition, a digital elevation model for Slovenia (EU-DEM) with 30 m resolution was used.

The ground truth data in our experiments were collected from the Slovenian land parcel identification system (LPIS). The original LPIS data consist of 177 different vegetation classes. These classes were grouped into 23 more general classes proposed by domain experts. The final data set includes 23 separate classes describing the type of farmland and one class describing all non-agricultural surfaces. Data have been collected for the year 2017.

**Figure 5:**
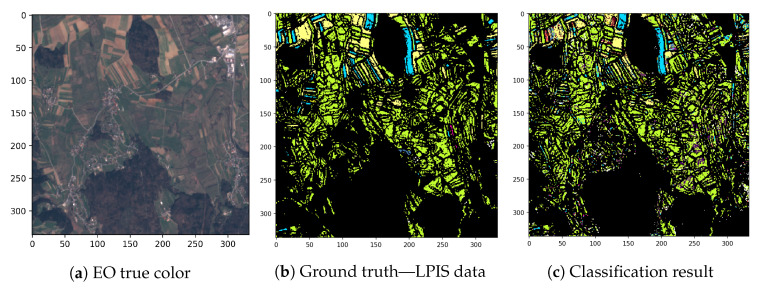
Partial input data, ground truth data and classification results.

A classification scenario is depicted in Figure 5. The figure shows a subset (true colour) of input EO data, the manually acquired ground truth data and the final automatic classification result of our algorithm. Based on the (easily obtainable EO data), the classification algorithm predicts ground-truth label (which is difficult to obtain and often contains incorrect data). When comparing Figure 5b,c, we observe the similarity between ground truth and the classification result.

### 4.2. Feature Engineering and Sampling

**Figure 6:**
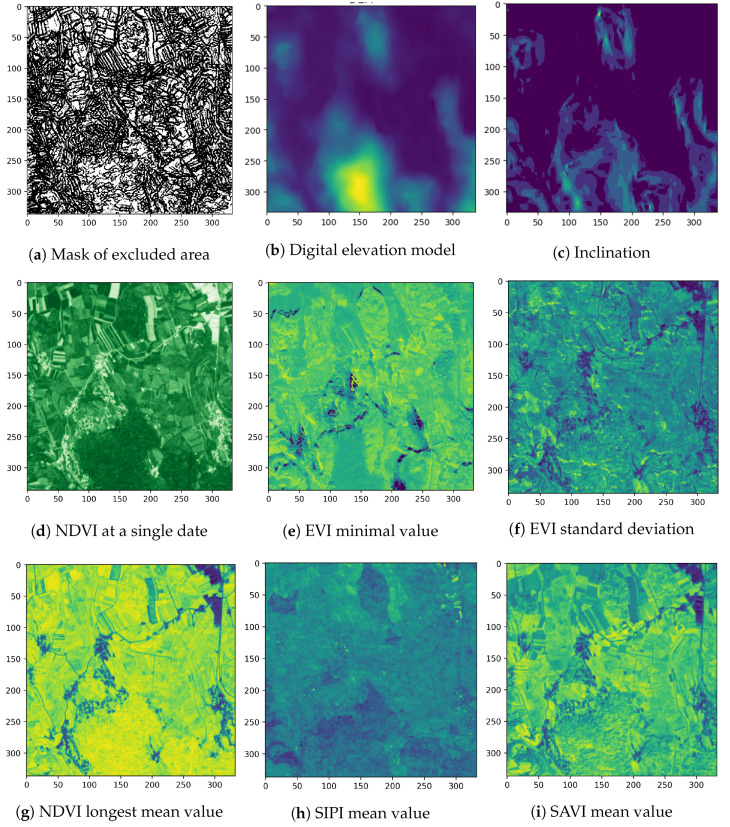
Examples of features derived from EO time series. Each feature represents a potentially significant parameter for land cover classification [20].

The EO data were collected for the entire year. Four raw band measurements (red, green, blue—RGB and near-infrared —NIR) and six relevant vegetation-related derived indices (normalized differential vegetation index—NDVI, normalized differential water index—NDWI, enhanced vegetation index—EVI, soil-adjusted vegetation index—SAVI, structure intensive pigment index—SIPI and atmospherically resistant vegetation index—ARVI) were considered. The derived indices are based on extensive domain knowledge and are used for assessing vegetation properties. In Figure 6d, an example of NDVI index is depicted, which is an indicator of vegetation health and biomass. Its value changes during the growing season of the plants and differs significantly from other non-planted areas. The NDVI is calculated as:

$$NDVI=\frac{NIR-red}{NIR+red}$$

Timeless features were extracted based on Valero et al. [20]. These features can describe the three most important crop stages: the beginning of greenness, the ripening period and the beginning of senescence [20,21]. Annual time series have different shapes due to the phenological cycle of a crop and characterize the evolution of a crop. With timeless features, they can be represented in a condensed form.

For each pixel, 18 features per each of the 10 time-series were generated. The raw value and maximum inclination for a given pixel were calculated from the elevation data as 2 additional features. In total, 182 features were used in the experiments.

The examples of learning features are depicted in Figure 6: EVI minimal value (Figure 6e), EVI standard deviation (Figure 6f), NDVI maximum mean value in a sliding temporal neighborhood of size 2 (Figure 6g), SIPI mean value (Figure 6h) and SAVI mean value (Figure 6i).

Prior to the experiments, edge detection [31] was performed on EO data, excluding the pixels at the borders of land plots. These pixels are potential mixed-class instances that can have a negative effect on the learning process. An example of an edge detection mask is depicted in Figure 6a. A balanced learning set was sampled from the entire data set with $20\times 10^{3}$ data points (pixels) representing each class. The classes with the lowest frequency were oversampled in the vicinity of sampled instances. The entire learning data set [32] consists of $480\times 10^{3}$ samples.

### 4.3. Feature Selection Benchmark Data Sets

Benchmark data sets for feature selection were taken from [6]. The description of all the data sets is given in Table 1. It is apparent from the table that our main data set, EOData [32] in the last row, differs from other data sets with its very high number of instances and relatively low number of features.

## 5. Results

### 5.1. Experimental Setup

To assess the quality of the selected feature subsets, a frontSurface metric was introduced to measure the surface under the Pareto front produced by the resulting subsets. With the analogy to the *area under a curve* (AUC), which is often used in the evaluation of classification methods, the frontSurface measure can also be called the *area under a front* (AUF). The area under a front can be interpreted as a measure of the efficiency of the feature selection algorithm. The surface is calculated as a simple sum of the best scores for each number of features *k*. In order to simplify the presentation and later evaluation, the surface is calculated only for a fixed maximum number of features *K* and then normalized. This makes it easier to compare fronts of different sizes and keeps them resistant to outliers with a large number of features. The measure is defined as 

$$\mathrm{AUF}:=\frac{\sum_{k=1}^{K}opt\left(k\right)}{K}\;\;opt\left(k\right):=\max_{|\mathrm{ITEM}|\leq k}\left\{item.result\right\},$$

where opt(k) represents the item with the highest score on the Pareto front, with a size of, at most, *k* (i.e., the best performing subset of features for a fixed number of features). In our case, the maximum number of features K=20 was selected because the selected feature subsets rarely have more than 20 features. Intuitively, the AUF measures the average of the best scoring items with a size of less than *K*.

All experiments on benchmarking data sets were performed with the same setup. The data for feature selection were first split into training and test subset (80:20) with stratified random split (sklearn.model_selection.train_test_split with a random state 2020 was used). The selected algorithm was run on a training subset, where the accuracy score was calculated as the $F_{1}$ score of the resulting model on a test set. For the FS-SDS algorithm, which uses internal data set splitting, the full data set was used. EO-data set was further split into two equal parts. The first part was used as a test set in the evaluation phase. The second part was used in the later analysis of the algorithm’s generalization in Section 6.2.

The resulting statistics were calculated using the best reported feature subset for a data set and feature selection algorithm. To partially avoid possible feature selection bias [33] (test data set is not separate from the data set used for feature selection), a set of 80:20 training/test balanced splits were done using random seeds from 20 to 50. For wrapping algorithms (which typically use a significant amount of time in the learning phase), it is often impossible to validate according the nested cross-validation loop strategy.

The final results were obtained as the AUF of $F_{1}$ score, produced by the models trained on training data sets using the selected feature subset and evaluated on corresponding test subsets. The results are used for comparative purposes between the different algorithms.

For FASTENER algorithm, the decision tree classifier provided by the scikit-learn library was used as the base classification algorithm (using Gini index for measuring quality of a split and a requirement of keeping, at a minimum, two examples in a split). A random mating pool of size 3 was selected and all item pairs were mated using information gain crossover. All items from the Pareto front were also carried over onto the next generation and mutated along with the newly created ones. For DT-forward, we used the same decision tree hyper parameters as with FASTENER. FS-SDS uses Extreme Learning Machine (ELM) for classification. The latter was configured with 160 hidden neurons and the sigmoid activation function.

### 5.2. Comparison with Similarity-Based Methods on EO Data

**Figure 7:**
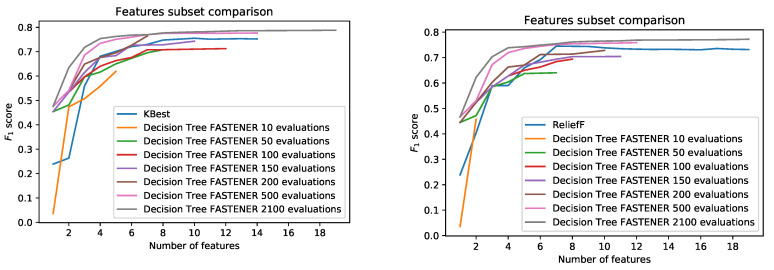
Pareto front comparison.

FASTENER results were compared with the results of the KBEST algorithm and the RELIEFF algorithm. Although outdated, they are presented in the literature as the currently best-performing methods in the field of land cover classification [22]. To no surprise, FASTENER achieves better results. Similar to FASTENER, the best feature subset for a fixed number of features was represented as an item in a Pareto front. The Pareto front visualization for reported optimal feature subsets is shown in Figure 7. The optimal feature subsets reported by the FASTENER algorithm outperform KBest and ReliefF. Since the tested implementation of ReliefF uses Python’s native KD tree, only 20% of the original training data was used to speed up the feature selection process in RELIEFF and compared with the subset selected by FASTENER on the same reduced training data.

### 5.3. Detailed Comparison with Wrapper Methods for EO Validation

Due to the nature of the EO data set (high number of instances), the evaluation of DT-forward, DT-backward, SVM-forward and SVM-backward [14] was not possible due to extremely long computation times. Along with FASTENER and POSS, we were also able to test the EO data set using FS-SDS [12]. The following evaluation presents a detailed comparison of POSS and FASTENER algorithms, and FS-SDS results are given at the end of the subsection.

**Figure 8:**
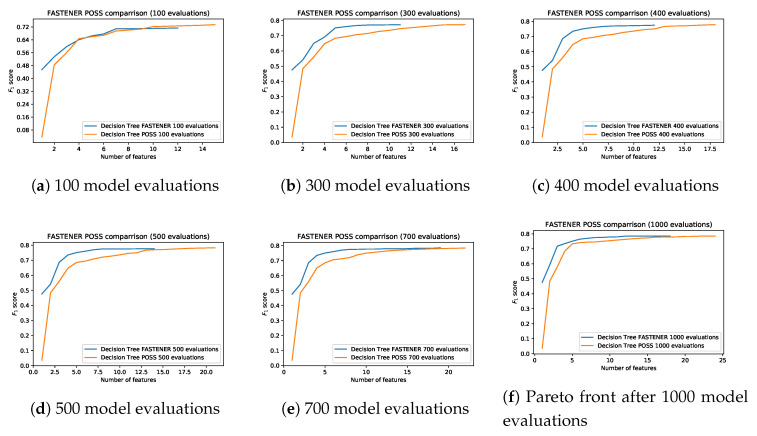
Comparison of the Pareto fronts produced by FASTENER and POSS algorithms after different numbers of iterations.

Figure 8 shows the progression of the Pareto front produced by POSS and FASTENER algorithms with the same number of model evaluations. It can be clearly seen that the convergence speed of the FASTENER algorithm outperforms the convergence speed of a comparable POSS implementation. Further analysis shows that the FASTENER algorithm converges about three-times faster than the POSS algorithm.

**Figure 9:**
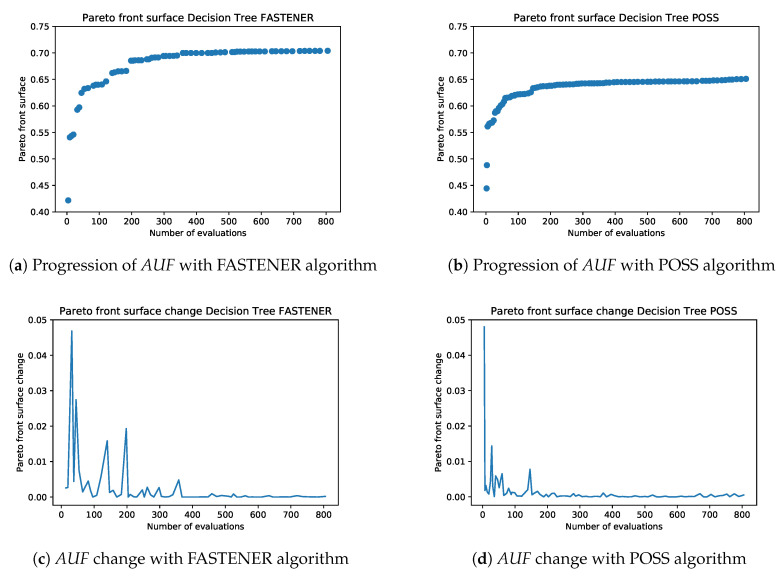
The comparison of the Pareto fronts generated by FASTENER and POSS algorithms after a different number of iterations. FASTENER exhibits better AUF scores and the discovery of several jumps, indicating the discovery of a distinct new best combination of features.

Comparisons of Pareto AUF and AUF changes for POSS and FASTENER algorithms during the first 800 iterations are shown in Figure 9. A quick visual comparison of the AUF graphs between POSS and the FASTENER results shows that the surface with the FASTENER algorithm is on average of 5% larger. The FASTENER AUF also exhibits much larger jumps. Larger jumps indicate a strong improvement in the $F_{1}$ score. This can either be a large improvement in the score for an existing *k* number of features or a slightly smaller improvement in the score for many smaller feature sets that dominate a large part of an existing Pareto front. Periodic larger jumps correspond to the invocation of purgeParetoFront routine, which tries to improve the results with additional information gain.

Another useful tool in convergence analysis is to compare the change in AUF presented in Figure 9c,d. The changes in the FASTENER algorithm are an indication of a higher convergence ratio. The computational complexity of AUF is minimal (linear in the size of the front) and can be easily computed while purging the Pareto front after each generation. The continuous calculation of the AUF (and its change) can be used as a helpful marker to measure the course of convergence in several of the above methods. In particular, the change in the area under a front can be used to modify the probabilities in the crossover and mutate methods to control the feature space search.

Detailed analysis of wrapper methods tested on EO-data is presented in Table 2. All the tested methods produce the area under a front with low variation, which can be attributed to a data set size. It can clearly be seen that FASTENER outperforms both POSS and FS-SDS.

### 5.4. Benchmark Data Set with Wrapper Methods

Along with FASTENER, forward decision tree selection method (DT-forward) [14] and FS-SDS algorithm [12] were tested for comparison on an open feature selection data sets [6] used for benchmarking.

Table 3 and Table 4 present comparisons of the FS-SDS algorithm, FASTENER and forward selection with decision tree (DT-forward). For each of the algorithms, 10 different data splits were used for feature selection and each feature subset was tested on 30 different test/train data splits. FASTENER was run with an exploration setting (larger generations) for 200 generations and the number of evaluations is a lot higher than in the EO setting. Each resulting Pareto front was purged, since adding more features might hurt performance on new data and the area under the Pareto front was calculated. Table 3 and Table 4 present mean, max, median and standard deviation of thus obtained surfaces under Pareto fronts, along with the number of model evaluations.

Table 3 shows that FASTENER outperforms FS-SDS on almost all data sets, except on the lung_discrete data set, where the mean and median AUF score are better with FS-SDS. Apart from outperforming score-wise, FASTENER also produces results faster (with fewer model evaluations) due to additional statistical data, while producing only marginal overhead due to initial entropy calculation.

Table 4 presents a comparison of features selected by FASTENER with features selected by forward selection with decision tree (DT-forward). DT-forward works by successively adding the best performing features, which can be suitable for small data sets, but the computational complexity explodes, as the number of features increases. As can be seen from Table 4 that FASTENER outperforms the DT-forward in a slightly less than half of the data sets. The difference between the maximum in DT-forward selection and FASTENER data in most of other cases is a few percentage points. The important improvement brought in by FASTENER is the efficiency of obtaining better (or just marginally worse) AUF with a lot fewer evaluations in comparisons to the forward selection. On average, the FASTENER uses between 2–5-times fewer model evaluations, depending on the number of features in the data set, while obtaining comparable or sometimes even better results. Additional insight presented by the experiment is the standard deviation when using different data splits. Standard deviation in smaller data sets is larger than in EO-data set, and a bit larger than the standard deviation using DT-forward since FASTENER includes additional random part during the algorithm run, while forward feature selection is deterministic.

Another FASTENER strength compared to the FS-SDS or DT-forward is the non-parametricity concerning the number of features. Both FS-SDS and DT-forward are rigid when selecting the feature subset size and increasing (in the case of forward selection) or changing (FS-SDS) subset size is computationally expensive. FASTENER automatically explores available search space unconstrained by the number of features, but they can be additionally constrained when reporting the results.

## 6. Discussion

### 6.1. Comparisons with other Methods

FASTENER builds on the idea of having multiple non-optimal items simultaneously considered for selection. The front in the main loop of the algorithm is a list of items for a fixed number of selected features. The additional parameter controls the size of the buckets, but initial testing concluded that increasing the bucket size to more than 2 decreased the performance and a more elitist parent selection (bucket sizes 1 and 2) was used. The exploration phase, where the population is allowed to deviate from the currently optimal front and only the items in the currently selected population are used as possible parents for a few rounds can be seen as a generalization of multiple fronts used in NSGA-II. The front purging after a selected number of rounds (controlled by a parameter) then returns the front to the current best and maintains an elitist gene selection.

Due to a large number of features and data points in the EO domain, smaller population sizes are more suitable for feature selection. In this case, the increase in bucket sizes (and non-optimal fronts) greatly increases the population size, which produces slower convergence as the elitism part does not kick in quickly enough.

Another specific thing about feature selection is the fact that the feature subset size dimension is discrete and mostly fully dense. Since the optimal sizes of subsets quickly converge even for a small number of selected features, the clustering optimization performed by NSGAA-II and RPSGAe does not improve the efficiency of convergence.

### 6.2. Generalization

**Figure 10:**
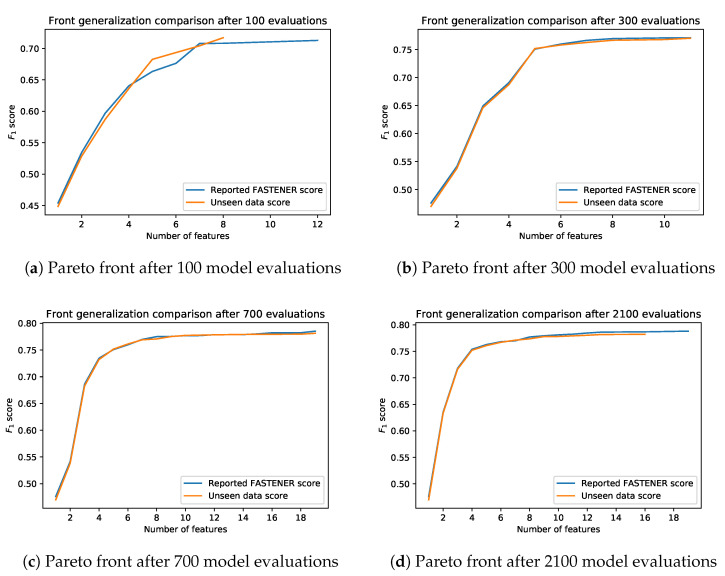
The generalization of results on unseen data presents small performance discrepancies between test data and previously unseen data.

An important aspect of feature subset optimization is further generalization to unseen data. As mentioned at the beginning of this section, an additional part of the data was kept unseen to the FASTENER algorithm during the phase of selecting the optimal subset of features. This hidden data set was used to analyze the overfitting of the FASTENER algorithm to the combination of training/test data. For each generation of the FASTENER algorithm, the features from the optimal items were used as features to train the model using the training data. However, this time the models were evaluated using the 10% of data not seen during FASTENER iterations. The overfitting was minimal and ranged from 0.5 to approximately 0.3 percentage points compared to the results on the test data set reported by the FASTENER algorithm. An important piece of information obtained from additional testing on the unseen data was the fact that some items from the reported optimal Pareto fronts were strictly dominated by feature subsets with fewer features. This was not surprising for subsets with a larger number of features, as the Pareto front “levels off” after a larger number of features. Results of the generalization analysis are presented in Figure 10.

**Figure 11:**
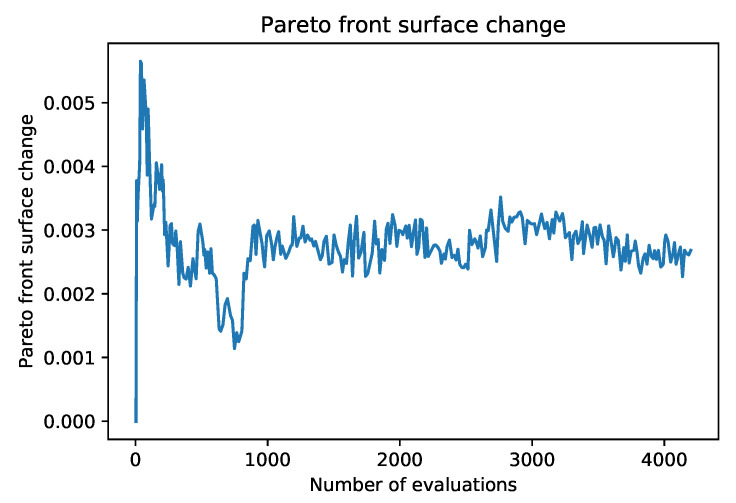
Low AUF difference between the test and unseen data shows good generalization abilities of the FASTENER algorithm.

The generalization on unseen data is quite good, which is at least partly due to a large data set compared to the number of algorithm iterations. The algorithm did not converge to some local optimum imposed by training data. Another way to analyze training performance on unseen data is to check the difference between AUF values on the reported front and unseen data. The plot of the AUF difference is shown in Figure 11. The difference levels off after approximately 60 iterations of the algorithm and appears to be constant with some variation.

**Figure 12:**
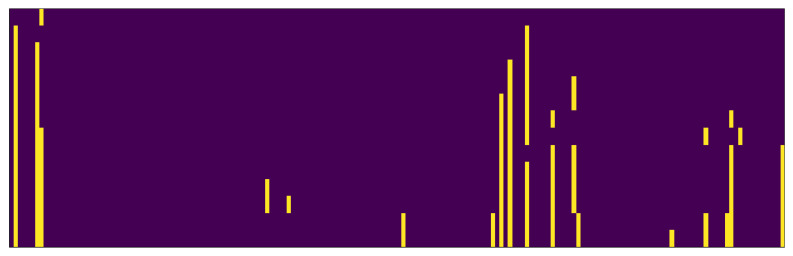
Visualization of optimal features for different feature subset sizes. The *x* axis represents the feature index, while the *y* axis depicts the number of features *k* (increasing with each row). FASTENER does not simply add new features as *k* is increased, but rather finds the best possible combination of features that gives the best possible classification result for a given *k*.

Apart from the generalization, it is interesting to observe the statistics for selected items and the statistics of individual genes during the execution of the algorithm. Figure 12 visualizes optimal selected features after the termination of the FASTENER algorithm. The yellow squares represent selected optimal features as genes. With *y* axis progression, new features are added. Interestingly, the algorithm does not simply extend the feature sets of *k* for the higher *k*’s, but rather examines previously excluded feature combinations that, with additional features, perform better.

**Figure 13:**
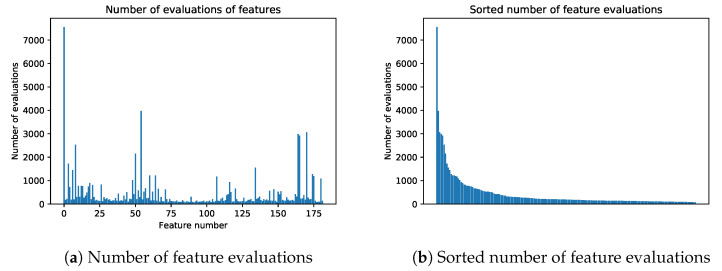
Number of feature evaluations. Although some features are not represented in Figure 12, they have often been evaluated by the algorithm.

Apart from selecting the optimal subsets, one should also look at the density of the different features, as they were involved during the iterations. The diagrams in Figure 13 show the number of evaluations of features during the run time of the algorithm. If the feature subset [1, 5, 7, 100] was evaluated, each of the features shown is considered to be evaluated once. Figure 13a shows the number of feature evaluations indexed by feature indices. The most evaluated feature is 0, since this was the only item in the starting Pareto front for the algorithm. Looking at other features, there are some areas of higher activity, but interestingly, while features between indices 50 and 75 seem to be fairly highly valued, they are very rarely included in the final optimal Pareto fronts. It seems that they are favored by heuristics. On the contrary, towards the end, features are fairly highly valued and are often present in optimal subsets.

## 7. Conclusions and Future Work

With the FASTENER algorithm, we have combined a wrapping methodology with information-theoretical measures based on entropy. The resulting feature selection method has proven to be very promising (superior in EO scenario), with its fast convergence (less learning iterations needed) and better accuracy (higher $F_{1}$ score and surface under the Pareto front) than the currently tested methods in the field. In the EO literature, feature selection methodologies have not been thoroughly explored and thus very basic algorithms, such as ReliefF and POSS have been reported to give the best results so far. We have also repeated experiments using FS-SDS, which FASTENER consistently outperformed. FASTENER was compared to FS-SDS and DT-forward algorithms on 25 open feature selection data sets, which are significantly different from the EO data set (a few instances and a lot of features). In terms of accuracy, FASTENER is comparable with DT-forward but generally achieves the same result with far fewer evaluations. Although the method was originally developed for applications within the EO scenarios, its usage in other domains seems promising. Any supervised machine learning problem (classification or regression) that requires optimization of the accuracy measure (either $F_{1}$ score or RMSE) with respect to the number of used features would benefit from the implementation of FASTENER.

Several aspects will be addressed in future work. Better theoretical justification of the algorithm is needed as well as the analysis of its convergence and other relevant properties. Possibilities of parallelization of FASTENER (similar as in [34] for POSS) should be examined to speed up the convergence.

Finally, for EO or similar problems, where the calculation of features themselves is computationally challenging, optimization of the final time of the learning process (including feature engineering and data acquisition) need to be performed.

## Figures and Tables

**Table 1:** Description of the data sets used for testing the FASTENER with number of instances, features and classes. I${}_{C↓}$ and I${}_{C↑}$ represent the percentage of instances representing the smallest and the largest class, respectively.

Data Set	Instances	Features	Classes	I${}_{C↓}$	I${}_{C↑}$
ALLAML	72	7129	2	35	65
arcene	200	10,000	2	44	56
BASEHOCK	1993	4862	2	50	50
CLL_SUB_111	111	11,340	3	10	46
COIL20	1440	1024	20	5	5
colon	62	2000	2	35	65
gisette	7000	5000	2	50	50
GLIOMA	50	4434	4	14	30
GLI_85	85	22,283	2	30	70
Isolet	1560	617	26	4	4
leukemia	72	7070	2	35	65
lung	203	3312	5	3	68
lung_discrete	73	325	7	7	29
lymphoma	96	4026	9	2	48
nci9	60	9712	9	3	15
ORL	400	1024	40	3	3
orlraws10P	100	10,304	10	10	10
PCMAC	1943	3289	2	50	50
Prostate_GE	102	5966	2	49	51
RELATHE	1427	4322	2	45	54
TOX_171	171	5748	4	23	26
USPS	9298	256	10	8	17
warpAR10P	130	2400	10	10	10
warpPIE10P	210	2420	10	10	10
Yale	165	1024	15	7	7
EOData	480,000	182	24	4	4

**Table 2:** Results of wrapper feature selection algorithms on EO data set. FASTENER yields the highest average AUF and lowest number of model evaluations. Standard deviation of the AUF is small for all methods. Best values accross different methods are bolded (in all tables).

Method	avg. AUF	sd	eval_n
POSS	0.66	0.0008	**1000**
FS-SDS	0.62	0.0008	120,000
FASTENER	**0.71**	0.0008	**1000**

**Table 3:** Mean, max, median value, standard deviation AUF and number of model evaluations. FS-SDS uses 30,000 model evaluations per each *k* (whereas for FASTENER the number of iterations produces the whole Pareto front) in the first iteration. Number of evaluations is reduced with the next iterations and stays in the range between (10,000–30,000).

	FS-SDS					FASTENER				
Data Set	Mean	Max	Medi	sd	eval_n*	Mean	Max	Medi	sd	eval_n
ALLAML	0.643	0.717	0.65	0.039	30,000	**0.776**	**0.894**	**0.78**	0.062	11,238
arcene	0.608	0.673	0.607	0.037	30,000	**0.707**	**0.785**	**0.713**	0.034	12,083
BASEHOCK	0.58	0.618	0.58	0.018	30,000	**0.742**	**0.78**	**0.744**	0.019	13,847
CLL_SUB_111	0.537	0.671	0.534	0.053	30,000	**0.626**	**0.79**	**0.623**	0.067	11,911
COIL20	0.655	0.673	0.657	0.01	30,000	**0.687**	**0.714**	**0.687**	0.012	14,214
colon	0.658	0.77	0.652	0.069	30,000	**0.763**	**0.9**	**0.757**	0.076	11,256
gisette	0.672	0.682	0.671	0.005	30,000	**0.786**	**0.802**	**0.786**	0.006	13,213
GLIOMA	0.549	0.687	0.561	0.067	30,000	**0.618**	**0.827**	**0.617**	0.095	11,674
GLI_85	0.659	0.746	0.665	0.063	30,000	**0.755**	**0.894**	**0.77**	0.065	11,238
Isolet	0.365	0.392	0.364	0.012	30,000	**0.396**	**0.432**	**0.395**	0.012	14,572
leukemia	0.62	0.77	0.618	0.06	30,000	**0.824**	**0.9**	**0.833**	0.057	11,248
lung	0.663	0.75	0.656	0.031	30,000	**0.727**	**0.828**	**0.731**	0.038	11,802
lung_discrete	**0.533**	0.666	**0.532**	0.068	30,000	0.511	**0.716**	0.516	0.086	11,753
lymphoma	0.539	0.619	0.548	0.058	30,000	**0.59**	**0.757**	**0.58**	0.07	11,719
nci9	0.331	0.513	0.311	0.097	30,000	**0.405**	**0.61**	**0.406**	0.088	12,081
ORL	0.347	0.395	0.351	0.029	30,000	**0.39**	**0.488**	**0.389**	0.04	13,540
orlraws10P	0.675	0.77	0.686	0.053	30,000	**0.694**	**0.821**	**0.695**	0.062	13,571
PCMAC	0.596	0.624	0.594	0.012	30,000	**0.71**	**0.736**	**0.708**	0.013	15,768
Prostate_GE	0.676	0.734	0.691	0.041	30,000	**0.748**	**0.837**	**0.749**	0.05	14,002
RELATHE	0.52	0.554	0.519	0.017	30,000	**0.667**	**0.707**	**0.667**	0.018	15,541
TOX_171	0.421	0.509	0.427	0.046	30,000	**0.553**	**0.647**	**0.55**	0.039	14,759
USPS	0.561	0.569	0.561	0.005	30,000	**0.583**	**0.593**	**0.583**	0.005	16,670
warpAR10P	0.502	0.588	**0.525**	0.052	30,000	**0.523**	**0.672**	0.518	0.065	13,840
warpPIE10P	0.582	0.646	0.589	0.037	30,000	**0.633**	**0.741**	**0.634**	0.041	13,630
Yale	0.38	0.454	0.376	0.035	30,000	**0.436**	**0.594**	**0.438**	0.052	15,307

**Table 4:** Mean, max, median value, standard deviation of AUF and number of model evaluations.

	DT Forward					FASTENER				
Data Set	Mean	Max	Medi	sd	eval_n	Mean	Max	Medi	sd	eval_n
ALLAML	**0.826**	0.893	**0.833**	0.044	106,830	0.776	**0.894**	0.78	0.062	11,238
arcene	0.659	0.76	0.665	0.054	149,895	**0.707**	**0.785**	**0.713**	0.034	12,083
BASEHOCK	**0.753**	**0.781**	**0.751**	0.015	72,825	0.742	0.78	0.744	0.019	13,847
CLL_SUB_111	0.54	0.655	0.54	0.049	169,995	**0.626**	**0.79**	**0.623**	0.067	11,911
COIL20	**0.707**	**0.729**	**0.706**	0.011	15,255	0.687	0.714	0.687	0.012	14,214
colon	0.751	0.888	0.745	0.066	29,895	**0.763**	**0.9**	**0.757**	0.076	11,256
gisette	**0.8**	**0.815**	**0.799**	0.007	74,895	0.786	0.802	0.786	0.006	13,213
GLIOMA	0.592	0.743	**0.626**	0.103	66,405	**0.618**	**0.827**	0.617	0.095	11,674
GLI_85	0.724	0.82	0.733	0.066	334,140	**0.755**	**0.894**	**0.77**	0.065	11,238
Isolet	**0.409**	**0.445**	**0.408**	0.014	9150	0.396	0.432	0.395	0.012	14,572
leukemia	**0.866**	**0.9**	**0.873**	0.03	105,945	0.824	0.9	0.833	0.057	11,248
lung	**0.76**	0.827	**0.761**	0.038	49,575	0.727	**0.828**	0.731	0.038	11,802
lung_discrete	**0.526**	0.689	**0.532**	0.08	4770	0.511	**0.716**	0.516	0.086	11,753
lymphoma	0.586	**0.785**	**0.581**	0.077	60,285	**0.59**	0.757	0.58	0.07	11,719
nci9	**0.419**	**0.64**	**0.414**	0.115	145,575	0.405	0.61	0.406	0.088	12,081
ORL	**0.407**	0.464	**0.41**	0.032	15,255	0.39	**0.488**	0.389	0.04	13,540
orlraws10P	**0.76**	**0.835**	**0.765**	0.055	154,455	0.694	0.821	0.695	0.062	13,571
PCMAC	**0.722**	**0.75**	**0.721**	0.013	49,230	0.71	0.736	0.708	0.013	15,768
Prostate_GE	**0.787**	**0.851**	**0.793**	0.041	89,385	0.748	0.837	0.749	0.05	14,002
RELATHE	**0.684**	**0.724**	**0.681**	0.016	64,725	0.667	0.707	0.667	0.018	15,541
TOX_171	0.469	0.536	0.466	0.035	86,115	**0.553**	**0.647**	**0.55**	0.039	14,759
USPS	0.564	0.574	0.565	0.005	3735	**0.583**	**0.593**	**0.583**	0.005	16,670
warpAR10P	**0.533**	0.659	**0.527**	0.06	35,895	0.523	**0.672**	0.518	0.065	13,840
warpPIE10P	**0.669**	**0.753**	**0.676**	0.033	36,195	0.633	0.741	0.634	0.041	13,630
Yale	0.407	0.505	0.406	0.048	15,255	**0.436**	**0.594**	**0.438**	0.052	15,307

## Abbreviations

The following abbreviations are used in this manuscript:

ARVI	Atmoshperically adjusted vegetation index
ASTFS	Automatic spectro-temporal feature selection
AUF	Area Under (Pareto) Front
DT	Decision Tree
EO	Earth Observation
ESA	European Space Agency
EVI	Enhanced Vegetation Index
FASTENER	Feature Selection Enabled by Entropy
LDA	Latent Dirichlet Allocation
LPIS	Land Parcel Identification System
NIR	Near Infra Red
NDVI	Normalized differential vegetation index
NDWI	Normalized differential water index
NLPCA	Non-linear Principal Components Analysis
PB	Petabyte
PCA	Principal Components Analysis
POSS	Pareto Optimization for Subset Selection
PPOSS	Parallel Pareto Optimization for Subset Selection
SAVI	Soil-adjusted Vegetation Index
FS-SDS	Feature Selection using Stohastic Diffucion Search
SIPI	Structure Insensitive Pigment Index
